# Supplementary material for: Evaluation of low-dose aspirin in the prevention of recurrent spontaneous preterm labour (the APRIL study): A multicentre, randomised, double-blinded, placebo-controlled trial
Source: PLoS Med. 2022 Feb 1;19(2):e1003892. doi: 10.1371/journal.pmed.1003892 (PMC8806064; doi:10.1371/journal.pmed.1003892)
Supplement: S1 Fig — (PDF) [file pmed.1003892.s001.pdf]

Figure S1 Maternal self-reported symptoms

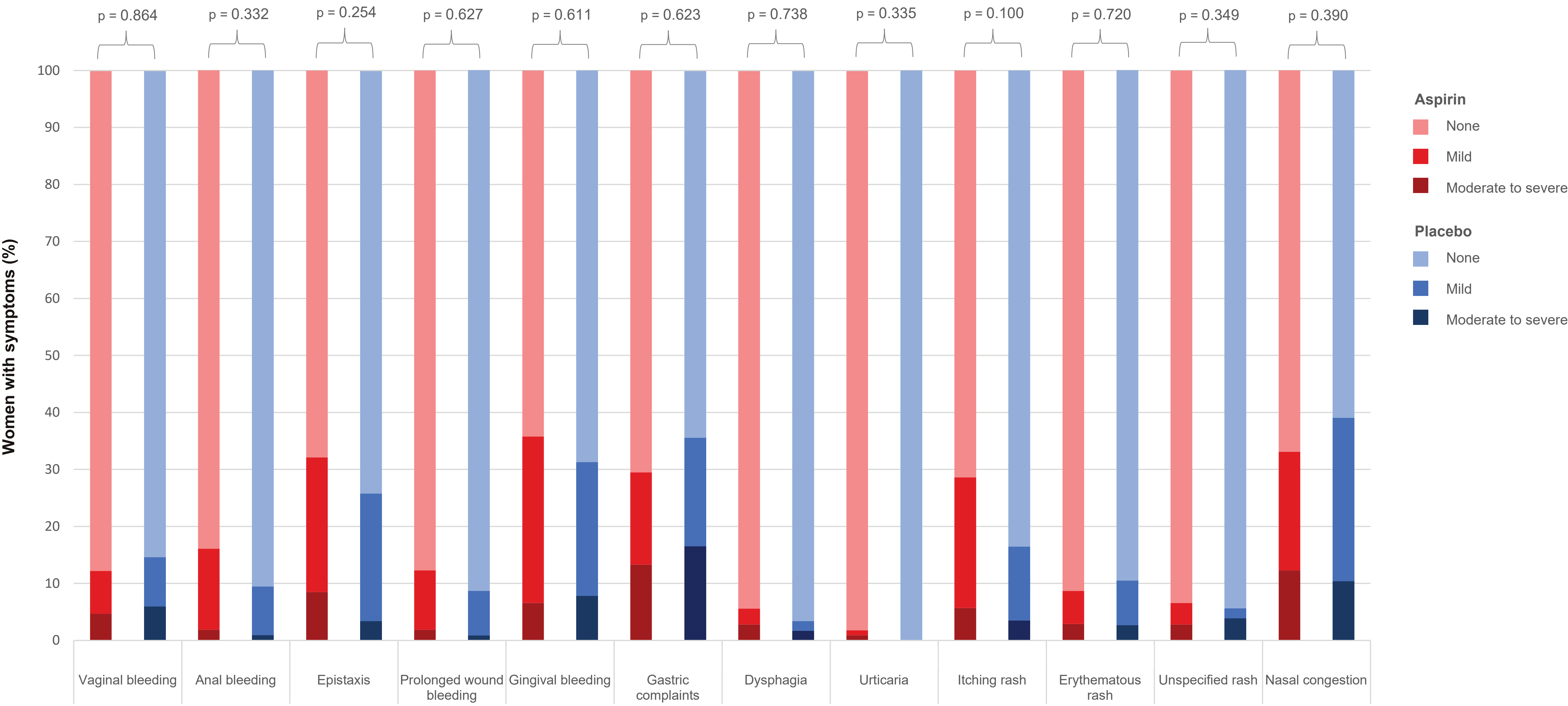

p-values were calculated with the chi-sqaure for trend
