# Supplementary material for: Evaluation of low-dose aspirin in the prevention of recurrent spontaneous preterm labour (the APRIL study): A multicentre, randomised, double-blinded, placebo-controlled trial
Source: PLoS Med. 2022 Feb 1;19(2):e1003892. doi: 10.1371/journal.pmed.1003892 (PMC8806064; doi:10.1371/journal.pmed.1003892)
Supplement: S1 Table — (PDF) [file pmed.1003892.s002.pdf]

Table S1 Inclusions per participating centre

	Aspirin (n=194)	Placebo (n=193)	Total n=387
**Tertiary care hospitals**			
Amsterdam UMC, location AMC	23	16	39
Amsterdam UMC, location VUmc	14	14	28
Leiden University Medical Centre	6	11	17
Maastricht University Medical Centre	1	0	1
Máxima Medical Centre	13	14	27
University Medical Centre Groningen	5	5	10
Radboud University Medical Centre	12	10	22
University Medical Centre Utrecht	11	14	25
**Secondary care hospitals**			
Amphia Hospital	11	14	25
Catharina Hospital	3	0	3
Deventer Hospital	7	8	15
Diakonessenhuis	5	7	12
Flevo Hospital Almere	7	7	14
Franciscus & Vlietland Hospital	3	1	4
Gelre Hospitals Apeldoorn	7	6	13
Haaglanden Medical Centre	4	6	10
Haga Hospital	7	8	15
Hospital Gelderse Vallei	0	3	3
Ikazia Hospital	2	1	3
Jeroen Bosch Hospital	1	2	3
Martini Hospital	10	6	16
Meander Medical Centre	4	3	7
Medical Spectrum Twente	0	1	1
OLVG	5	10	15
Ommelander Hospital Group	2	1	3
Reinier de Graaf Hospital	2	4	6
Spaarne Hospital	4	5	9
Sint Antonius Hospital	1	2	3
Tergooi Hospital	7	3	10
Treant Care Group, Bethesda Hospital	3	2	5
VieCuri Medical Centre	1	2	3
Wilhelmina Hospital	1	1	2
Hospital Group Twente Almelo	10	4	14
Zuyderland Medical Centre	2	2	4
