# Supplementary material for: Evaluation of low-dose aspirin in the prevention of recurrent spontaneous preterm labour (the APRIL study): A multicentre, randomised, double-blinded, placebo-controlled trial
Source: PLoS Med. 2022 Feb 1;19(2):e1003892. doi: 10.1371/journal.pmed.1003892 (PMC8806064; doi:10.1371/journal.pmed.1003892)
Supplement: S2 Table — (PDF) [file pmed.1003892.s003.pdf]

Table S2 Medication use according to treatment group

	**Aspirin (n = 194)**	**Placebo (n = 193)**	**p-value**
**Initiation study medication –** median (IQR)	14^{+5} (13^{+3}-15^{+6})	15^{+1} (12^{+6}-16^{+0})	0.359[a]
**Adherence** (%) – median (IQR)	97.4 (90.9-99.3)	95.7 (86.4-99.4)	0.133[a]
Adherence ≥80%	125 (64.4%)	121 (62.7%)	
Non-adherent (<80%)	19 (9.8%)	24 (12.4%)	0.524[b]
Unknown adherence	50 (25.8%)	48 (24.9%)	

[a] Mann Withney U test

[b] Chi-square test
