# Supplementary material for: Evaluation of low-dose aspirin in the prevention of recurrent spontaneous preterm labour (the APRIL study): A multicentre, randomised, double-blinded, placebo-controlled trial
Source: PLoS Med. 2022 Feb 1;19(2):e1003892. doi: 10.1371/journal.pmed.1003892 (PMC8806064; doi:10.1371/journal.pmed.1003892)
Supplement: S3 Table — (PDF) [file pmed.1003892.s004.pdf]

Table S3 Additional neonatal outcomes according to treatment group

	**Aspirin**<br><br>**(n = 194)**	**Placebo**<br><br>**(n = 193)**
Number of days on ventilation support (intubation or CPAP)	0.95 (±5.2)	0.82 (± 5.9)
IRDS requiring surfactant therapy	5 (2.6%)	7 (3.6%)
Patent ductus arteriosus (PDA) requiring treatment	1 (0.5%)	2 (1.0%)
Hypotension requiring inotropics	1 (0.5%)	2 (1.0%)
Intestinal perforation	0 (0%)	0 (0%)
Convulsions	0 (0%)	0 (0%)
Asphyxia	4 (2.1%)	1 (0.5%)
Pneumothorax/pneumomediastinum	0 (0%)	3 (1.6%)
Early onset neonatal sepsis, culture proven	0 (0%)	2 (1.0%)
Late onset neonatal sepsis, culture proven	4 (2.1%)	1 (0.5%)
Neonatal meningitis	0 (0%)	0 (0%)
Cerebellar bleeding	1 (0.5%)	0 (0%)
Cephalic hematoma	1 (0.5%)	0 (0%)
Other cerebral bleeding [a]	4 (2.1%)	0 (0%)
Gastrointestinal bleeding	0 (0%)	0 (0%)
Lung bleeding	0 (0%)	0 (0%)
Other bleeding	0 (0%)	0 (0%)

[a] 3 germinal matrix hematomas, 1 possible epidural bleed (or artefact from cephalic hematoma)
