# Supplementary material for: Evaluation of low-dose aspirin in the prevention of recurrent spontaneous preterm labour (the APRIL study): A multicentre, randomised, double-blinded, placebo-controlled trial
Source: PLoS Med. 2022 Feb 1;19(2):e1003892. doi: 10.1371/journal.pmed.1003892 (PMC8806064; doi:10.1371/journal.pmed.1003892)
Supplement: S4 Table — (PDF) [file pmed.1003892.s005.pdf]

Table S4 List of Serious Adverse Events

Description of event	Aspirin (n = 204)	Placebo (n = 202)
**Maternal serious adverse events**		
Blood loss (1L) at cerclage placement	1	0
Mild pericarditis	0	1
Infected placental remnants requiring repeated curettage and blood transfusions	1	0
**Fetal chromosomal abnormalities**		
Leber congenital amaurosis	0	1
Trisomy 21 [a]	1	2
Duplication 7p22.1 [a]	1	0
Trisomy 13 [a]	0	1
Omphalocele + Beckwith-Wiedemann Syndrome [a]	1	0
**Fetal structural defects**		
Ventricular septal defect [a]	1	0
Swollen and blue discolouration of labia	0	1
Hypoplastic left heart syndrome [a]	0	1
Bilateral vocal cord paresis	1	0
Glandular hypospadia	0	1
Patent canal of Nuck, herniation of right adnex	0	1
Hydrocele testis	1	0
Congenital cystic adenomatoid malformation lung [a]	1	0
Talipes equinovarus	1	0
Bilateral hydronephrosis, resolved spontaneously	1	0
**Neonatal disease**		
Staphylococcal scalded skin syndrome	0	1
Asymptomatic cytomegalovirus infection	0	1
**At least one serious adverse event, no. (%)**	11 (5.4%)	11 (5.4%)
**Number of serious adverse events, no (%)**	11 (5.4%)	11 (5.4%)

[a] excluded from the trial analyses
