# Supplementary material for: Evaluation of low-dose aspirin in the prevention of recurrent spontaneous preterm labour (the APRIL study): A multicentre, randomised, double-blinded, placebo-controlled trial
Source: PLoS Med. 2022 Feb 1;19(2):e1003892. doi: 10.1371/journal.pmed.1003892 (PMC8806064; doi:10.1371/journal.pmed.1003892)
Supplement: S5 Table — (PDF) [file pmed.1003892.s006.pdf]

**Table S5** Pre-specified subgroup analyses

| | Aspirin Preterm birth <37 weeks | | Placebo Preterm birth <37 weeks | | Relative Risk [b] (95% CI) | p-value subgroup | p-value interaction term [c] |
|---|---|---|---|---|---|---|---|
| | n | % | n | % | | | |
| **Initiation of treatment** | | | | | | | |
| $8^{+0}$ - $11^{+6}$ weeks | 7/25 | 28.0% | 9/30 | 30.0% | 0.93 (0.41-2.15) | 0.871 | 0.664 |
| $12^{+0}$ - $16^{+0}$ weeks | 31/157 | 19.7% | 39/150 | 26.0% | 0.76 (0.50-1.15) | 0.194 | |
| **Progesterone treatment** | | | | | | | |
| Progesterone | 31/136 | 22.8% | 39/129 | 30.2% | 0.75 (0.50-1.13) | 0.810 | 0.406 |
| No progesterone | 10/58 | 17.2% | 10/64 | 15.6% | 1.10 (0.50-2.46) | 0.172 | |
| **Cervical length screening ($14^{+0}$-$23^{+6}$ weeks)** | | | | | | | |
| < 25 mm | 4/12 | 33.3% | 5/8 | 62.5% | 0.53 (0.20-1.40) | 0.201 | 0.422 |
| ≥ 25 mm | 35/157 | 22.3% | 39/143 | 27.3% | 0.82 (0.55-1.22) | 0.319 | |
| **Gestational age of a previous preterm birth [a]** | | | | | | | |
| < $30^{+0}$ weeks | 15/78 | 19.2% | 28/86 | 32.6% | 0.59 (0.34-1.02) | 0.059 | 0.042 |
| $30^{+0}$ - $33^{+6}$ weeks | 17/67 | 25.4% | 18/63 | 28.6% | 0.89 (0.50-1.57) | 0.681 | |
| $34^{+0}$ - $36^{+6}$ weeks | 9/49 | 18.4% | 3/44 | 6.8% | 2.69 (0.78-9.32) | 0.118 | |
| **Onset of previous spontaneous preterm birth [a]** | | | | | | | |
| Spontaneous contractions with intact membranes | 13/82 | 15.9% | 12/76 | 15.8% | 1.00 (0.49-2.06) | 0.991 | 0.573 |
| Preterm prelabour rupture of membranes | 28/112 | 25.0% | 37/117 | 31.6% | 0.79 (0.52-1.20) | 0.269 | |

[a] If women had multiple prior preterm births, they were classified according to the earliest preterm birth
[b] Relative risks and the corresponding p-values were calculated using generalised linear regression analysis
[c] Statistical significance of the subgroups was calculated by adding an interaction term to the model
