# Supplementary material for: Evaluation of low-dose aspirin in the prevention of recurrent spontaneous preterm labour (the APRIL study): A multicentre, randomised, double-blinded, placebo-controlled trial
Source: PLoS Med. 2022 Feb 1;19(2):e1003892. doi: 10.1371/journal.pmed.1003892 (PMC8806064; doi:10.1371/journal.pmed.1003892)
Supplement: S1 Appendix — (PDF) [file pmed.1003892.s007.pdf]

Appendix S1 Comparison to study protocol

Minor changes were made as compared to the published protocol and these are listed below:

- The study protocol states that the independent Data Safety Monitoring Committee would monitor the patient safety every 6 months, starting 9 months after the inclusion of the first patient. However, due to a rather slow inclusion rate the first months of the trial, the committee decided to perform monitoring every 100 inclusions (with completed follow-up).

- Centiles of birth weight will now be calculated based on the birthweight chart by Hoftiezer et al. This birth weight chart was not yet available at the time of the production and publication of the study protocol. However, the birth weight chart by Hoftiezer et al. is currently most accepted for the calculation of growth centiles in Dutch practice[1]. Growth restriction was still defined as growth below the 10th centile.

- In the published APRIL study protocol we planned to analyse preterm birth rates ≤28, ≤32 and ≤34 weeks of gestation. During the establishment of this analysis plan the research team decided to limit the analyses to ≤28 and ≤34 weeks of gestation to reduce multiple testing. Preterm births ≤28, ≤34 weeks and ≤37 weeks of gestation will be analysed as a group and also separately based on the onset of birth: spontaneous or indicated.

References

1. Hoftiezer L, Hof MHP, Dijs-Elsinga J, Hogeveen M, Hukkelhoven C, van Lingen RA. From population reference to national standard: new and improved birthweight charts. Am J Obstet Gynecol. 2019;220(4): 383 e381-383 e317.
