# Supplementary material for: Evaluation of low-dose aspirin in the prevention of recurrent spontaneous preterm labour (the APRIL study): A multicentre, randomised, double-blinded, placebo-controlled trial
Source: PLoS Med. 2022 Feb 1;19(2):e1003892. doi: 10.1371/journal.pmed.1003892 (PMC8806064; doi:10.1371/journal.pmed.1003892)
Supplement: S3 Appendix — (PDF) [file pmed.1003892.s009.pdf]

Appendix S3 Definitions of outcomes

Definitions of birth outcomes

Outcome	Definition
**Preterm birth**	Birth from 16^{+0} to 36^{+6} weeks of gestation.
Spontaneous onset	Preterm birth following either the spontaneous onset of contractions with intact membranes or prelabor rupture of membranes
Indicated	Medically induced labor or a primary caesarean delivery because of a maternal or fetal complication.
**Midtrimester fetal loss**	Fetal loss from 16^{+0} to 21^{+6} weeks of gestation after spontaneous contractions or spontaneous rupture of the membranes.
**Small for gestational age**	Birthweight <10th percentile for the gestational age using the birthweight chart by Hoftiezer et al.[1]

Definitions of neonatal outcomes

Outcome	Definition
**Asphyxia**	Diagnosed in case of an Apgar score after 5 minutes ≤5 or resuscitation/ventilation for 10 minutes after birth or pH<7.0 and base excess >16 mmol/L (fetal blood taken form the umbilical cord).[2]
**BPD**	Infants receiving supplemental oxygen for at least 28 days at 36 weeks postmenstrual age. Classification will take place using an oxygenreductiontest.[3]
**Culture proven sepsis**	Diagnosed by the combination of clinical signs of infection and positive blood cultures. Distinction will be made between:
Early onset sepsis (EONS)	clinical suspicion and positive blood culture <72 hours.
Late onset sepsis (LONS)	clinical suspicion, positive blood culture and a CRP blood level of >10mg/L >72 hours.[4]
**IVH > grade 2**<br>**PVL > grade 1**	Diagnosed by repeated neonatal cranial ultrasound by a neonatologist according to the guidelines on neuroimaging of the Papille classification described by de Vries and Ment et al.[5,6]
**NEC > stage 1**	Diagnosis according to Bell staging criteria.[7]
**Pneumothorax**	Visible on chest x-ray and/or the need to perform drainage.
**Proven meningitis**	Clinical suspicion of meningitis supported by positive liquor culture or liquor analysis highly suggestive for meningitis.
**Retinopathy of prematurity**	grading following the international classification of retinopathy of prematurity.[8,9]

**Mortality**	Death of a fetus or neonate at any time between a gestational age ≥16 weeks and discharge.
Fetal death	Death during pregnancy (≥16 weeks) or during labor.
Neonatal death	Death occurring in the period after birth until discharge.

Definitions of maternal outcomes

Outcome	Definition
**Eclampsia**	Seizures in a pregnancy complicated by preeclampsia.
**Gestational diabetes**	Onset of diabetes after 20 weeks gestational age.
**HELLP-syndrome**	Combination of hemolysis, elevated liver enzymes and thrombocytopenia with or without the presence of proteinuria or hypertension.
**Hospital admissions for threatened preterm labor**	Number and percentage of women with an admission for threatened preterm labor.
**Hospital admissions for vaginal bleeding**	Number and percentage of women with an admission for vaginal bleeding during pregnancy.
**Hospital admissions for any reason**	Total days admitted to a hospital for any reason
**Maternal infection**	Clinical diagnosis of urinary or genital tract infections and treated with antibiotics.
**Maternal mortality**	Death up to 28 days post estimated delivery date.
**Maternal self-reported symptoms**	Women receive a diary to score the following complaints: vaginal bleeding/spotting, anal blood loss, epistaxis, prolonged wound bleeding, gingival bleeding, gastric complaints, trouble swallowing, urticaria, erythema, itching rash, unspecified rash and nasal congestion. Women could score these complaints from 0-5 and indicate at which gestational age they experienced the complaints. The scores in the diary were defined as follows:<br>0 = none<br>1 = little imposition<br>2 = some imposition<br>3 = imposition<br>4 = a lot of imposition<br>5 = unbearable imposition<br><br>For analysis we redefined the scores to the following categories:<br>No symptoms = 0<br>Mild symptoms = 1+2<br>Moderate to severe = 3+4+5
**Placental abruption**	Clinical diagnosis of (partial) detachment of the placenta from the uterine wall.

**Preeclampsia**	Pregnancy-induced hypertension in combination with proteinuria (≥300 mg in 24 hours) or thrombocytopenia (<150 x10e9/L), impaired liver function or renal insufficiency. This may occur during pregnancy or the postpartum period.[10]
**Pregnancy-induced hypertension**	New onset of hypertension (≥ 140 mmHg systolic and/or ≥90 mmHg diastolic blood pressure) after 20 weeks of gestation measured on at least two occasions four hours apart.[10]
**Pulmonary edema**	Based on clinical findings.
**Thromboembolic disease**	Deep vein thrombosis or pulmonary embolism.
**Tocolytic therapy**	Received tocolytic therapy during pregnancy for the delay/prevention of spontaneous preterm birth.

REFERENCES

1. Hoftiezer L, Hof MHP, Dijs-Elsinga J, Hogeveen M, Hukkelhoven C, van Lingen RA. From population reference to national standard: new and improved birthweight charts. Am J Obstet Gynecol 2019; 220(4): 383 e1–e17.
2. Cornette L. Therapeutic hypothermia in neonatal asphyxia. Facts Views Vis Obgyn 2012; 4(2): 133–9.
3. Jobe AH, Bancalari E. Bronchopulmonary dysplasia. Am J Respir Crit Care Med 2001; 163(7): 1723–9.
4. Stoll BJ, Hansen N, Fanaroff AA, et al. Late-onset sepsis in very low birth weight neonates: the experience of the NICHD Neonatal Research Network. Pediatrics 2002; 110(2 Pt 1): 285–91.
5. de Vries LS, Liem KD, van Dijk K, et al. Early versus late treatment of posthaemorrhagic ventricular dilatation: results of a retrospective study from five neonatal intensive care units in The Netherlands. Acta Paediatr 2002; 91(2): 212–7.
6. Ment LR, Bada HS, Barnes P, et al. Practice parameter: neuroimaging of the neonate: report of the Quality Standards Subcommittee of the American Academy of Neurology and the Practice Committee of the Child Neurology Society. Neurology 2002; 58(12): 1726–38.
7. Kliegman RM, Walsh MC. Neonatal necrotizing enterocolitis: pathogenesis, classification, and spectrum of illness. Curr Probl Pediatr 1987; 17(4): 213–88.
8. An international classification of retinopathy of prematurity. The Committee for the Classification of Retinopathy of Prematurity. Arch Ophthalmol 1984; 102(8): 1130–4.
9. An international classification of retinopathy of prematurity. II. The classification of retinal detachment. The International Committee for the Classification of the Late Stages of Retinopathy of Prematurity. Arch Ophthalmol 1987; 105(7): 906–12.
10. Report of the American College of Obstetricians and Gynecologists' Task Force on Hypertension in Pregnancy. Obstet Gynecol 2013; 122(5): 1122–31.
