# Supplementary material for: Evaluation of low-dose aspirin in the prevention of recurrent spontaneous preterm labour (the APRIL study): A multicentre, randomised, double-blinded, placebo-controlled trial
Source: PLoS Med. 2022 Feb 1;19(2):e1003892. doi: 10.1371/journal.pmed.1003892 (PMC8806064; doi:10.1371/journal.pmed.1003892)
Supplement: S4 Appendix — (PDF) [file pmed.1003892.s010.pdf]

Appendix S4 Calculation of medication adherence

For calculation of the expected doses per patient, we used the patient reported gestational age of therapy initiation. In case reported initiation was later than 16^{+0} gestational age, than 16^{+0} was used for calculation. If the exact gestational age at initiation of therapy was unknown, it was estimated based on randomization date. On average, medication delivery at the patient's address was five days after randomization.

The expected medication use was calculated as follows:

Expected doses per patient = **35^{+6} gestational age** *or* **gestational age in case of preterm birth** *(whichever comes first)* − **gestational age at medication delivery***

** Gestational age at randomisation + 3 days*

Women were supplied with diaries to record on which days they took the study medication. In addition, women were required to return all leftover medication after completion or cessation of therapy for pill counts. The diaries and leftover medication were be used to make an estimate of study medication used by each woman. In case of discrepancies on the used medication between the diary and the pill counts, the lowest number of use, leading to the lowest adherence rate, was used.

Medication adherence was calculated as follows:

$$\text{Adherence \%} = \frac{\text{Number of doses of study medication used}}{\text{Expected number of doses for each patient}}$$

Women were considered adherent when they took the medication ≥80% of the days they should have taken it. Women who did not return their medication diary or leftover study medication had unknown medication adherence and were excluded from the sensitivity analysis.
