# Supplementary material for: Evaluation of low-dose aspirin in the prevention of recurrent spontaneous preterm labour (the APRIL study): A multicentre, randomised, double-blinded, placebo-controlled trial
Source: PLoS Med. 2022 Feb 1;19(2):e1003892. doi: 10.1371/journal.pmed.1003892 (PMC8806064; doi:10.1371/journal.pmed.1003892)
Supplement: S5 Appendix — (PDF) [file pmed.1003892.s011.pdf]

APRIL

STATISTICAL ANALYSIS PLAN

Study title: **Low dose Aspirin for the Prevention of Recurrent Spontaneous Preterm Birth – a randomised double blind placebo controlled trial**

Short title: APRIL
EudraCT: 2015-003220-31
Dutch Trial Register: NTR 5675

Funded by: ZonMw

Version: 1.0 Date: 1 Aug 2020

		Signature:	Date:
Prepared by:	A.J.E.M.C. Landman, MD<br>Coordinating researcher<br>Department of Obstetrics & Gynecology<br>Amsterdam UMC, location VUmc	[illegible]	3 aug 2020
	C. Naaktgeboren, PhD<br>Consultant Statician<br>Department of Obstetrics & Gynecology<br>Amsterdam UMC, location AMC		
	M.C. Jansen-van der Weide, PhD<br>Consultant Statician<br>Department of Obstetrics & Gynecology<br>Amsterdam UMC, location AMC		
Approved by:	M.A. Oudijk, MD, PhD<br>Perinatologist<br>Department of Obstetrics & Gynecology<br>Amsterdam UMC, location AMC	[illegible]	3 aug 2020.
	M.A. de Boer, MD, PhD<br>Perinatologist<br>Department of Obstetrics & Gynecology<br>Amsterdam UMC, location VUmc	[illegible]	4/8/'20.
	Prof. C.J.M. de Groot, MD, PhD<br>Perinatologist<br>Department of Obstetrics & Gynecology<br>Amsterdam UMC	[illegible]	4/8/20

STATISTICAL ANALYSIS PLAN

Study title:	**Low dose Aspirin for the Prevention of Recurrent Spontaneous Preterm Birth – a randomised double blind placebo controlled trial**
Short title:	APRIL
EudraCT:	2015-003220-31
Dutch Trial Register:	NTR 5675
Funded by:	ZonMw
Version:	1.0 Date: 1 Aug 2020

		Signature:	Date:
Prepared by:	A.J.E.M.C. Landman, MD<br>Coordinating researcher<br>Department of Obstetrics & Gynecology<br>Amsterdam UMC, location VUmc		
	C. Naaktgeboren, PhD<br>Consultant Statician<br>Department of Obstetrics & Gynecology<br>Amsterdam UMC, location AMC		
	M.C. Jansen-van der Weide, PhD<br>Consultant Statician<br>Department of Obstetrics & Gynecology<br>Amsterdam UMC, location AMC	[signature]	18-8-2020
Approved by:	M.A. Oudijk, MD, PhD<br>Perinatologist<br>Department of Obstetrics & Gynecology<br>Amsterdam UMC, location AMC		
	M.A. de Boer, MD, PhD<br>Perinatologist<br>Department of Obstetrics & Gynecology<br>Amsterdam UMC, location VUmc		
	Prof. C.J.M. de Groot, MD, PhD<br>Perinatologist<br>Department of Obstetrics & Gynecology<br>Amsterdam UMC		

STATISTICAL ANALYSIS PLAN

Study title:	**Low dose Aspirin for the Prevention of Recurrent Spontaneous Preterm Birth – a randomised double blind placebo controlled trial**
Short title:	APRIL
EudraCT:	2015-003220-31
Dutch Trial Register:	NTR 5675
Funded by:	ZonMw
Version:	1.0 Date: 1 Aug 2020

		Signature:	Date:
Prepared by:	A.J.E.M.C. Landman, MD<br>Coordinating researcher<br>Department of Obstetrics & Gynecology<br>Amsterdam UMC, location VUmc	________________________	____________
	C. Naaktgeboren, PhD<br>Consultant Statician<br>Department of Obstetrics & Gynecology<br>Amsterdam UMC, location AMC	Christiana Naaktgeboren	03-08-2020
	M.C. Jansen-van der Weide, PhD<br>Consultant Statician<br>Department of Obstetrics & Gynecology<br>Amsterdam UMC, location AMC	________________________	____________
Approved by:	M.A. Oudijk, MD, PhD<br>Perinatologist<br>Department of Obstetrics & Gynecology<br>Amsterdam UMC, location AMC	________________________	____________
	M.A. de Boer, MD, PhD<br>Perinatologist<br>Department of Obstetrics & Gynecology<br>Amsterdam UMC, location VUmc	________________________	____________
	Prof. C.J.M. de Groot, MD, PhD<br>Perinatologist<br>Department of Obstetrics & Gynecology<br>Amsterdam UMC	________________________	____________

TABLE OF CONTENTS

LIST OF ABBREVIATIONS 3

1. INTRODUCTION 4

1.1. Study background 4

1.2. Study objectives 4

1.3. Study design 4

1.4. Sample size and power 4

1.5. Study population 4

1.5.1. Inclusion criteria 4
1.5.2. Exclusion criteria 4

1.6. Randomisation and masking 5

1.7. Ethical approval and consent 5

1.7.1. Regulation statement 5
1.7.2. Consent 5

1.8. Treatment of subjects 5

1.8.1. Investigational product 5
1.8.2. Use of co-intervention 5

2. STATISTICAL ANALYSIS 6

2.1. Trial profile 6

2.2. Analysis populations 6

2.2.1. Intention to treat (ITT) population 6
2.2.2. Per protocol population 6

2.3. Baseline characteristics 6

2.4. Primary outcome 6

2.5. Secondary outcomes 7

2.5.1. Neonatal outcomes 7
2.5.2. Birth outcomes 7
2.5.3. Maternal outcomes 7
2.5.4. Additional outcomes in supplementary information 7

2.6. Subgroup analyses 8

2.7. Safety outcomes 8

2.7.1. Treatment compliance 8
2.7.2. Premature discontinuation of study medication 9
2.7.3. Adverse events 9

2.8. Statistical analyses 9

2.9. Data management and reproducibility of the analyses 10

2.10. Missing data 10

2.11. Interim Analyses and Data Monitoring 11
4. COMPARISON TO STUDY PROTOCOL 12
5. MOCK TABLES AND FIGURES 13
5.1. Flow diagram of participants 13
5.2. Baseline characteristics 14
5.3. Primary and secondary outcomes 16
5.3.1. Birth outcomes 16
5.3.2. Kaplan-Meier plot 17
5.3.3. Neonatal outcomes 18
5.3.4. Maternal outcomes 19
5.4. Subgroup analyses 20
6. SUPPLEMENTARY TABLES 21
6.1. Additional neonatal outcomes 21
6.2. Additional maternal outcomes 22
6.3. Reasons for premature discontinuation of study medication 23
6.4. List of Serious Adverse Events (SAEs) 23
6.5. Number of inclusions per participating center 24
7. DEFINITIONS OF OUTCOMES 25
7.1. Definitions of birth outcomes 25
7.2. Definitions of neonatal outcomes 25
7.3. Definitions of maternal outcomes 26
REFERENCES 27

List of Abbreviations

BPD -	Bronchopulmonary dysplasia
CPAP -	Continuous Positive Airway Pressure
EONS -	Early onset sepsis
ITT -	Intension To Treat
IRDS -	Infant respiratory distress syndrome
IVH -	Intraventricular haemorrhage
LONS -	Late onset sepsis
NEC -	Necrotizing enterocolitis
PPROM -	Premature prelabour rupture of membranes
PVL -	Periventricular leukomalacia
ROP -	Retinopathy of premature

1. Introduction

1.1. Study background

A detailed study background is provided in the published study protocol.[1]

We hypothesize that low-dose aspirin started before 16 weeks gestation will reduce the rate of preterm birth. To test this hypothesis we are performing the APRIL study: Low dose Aspirin for the Prevention of Recurrent Spontaneous Preterm Birth. This is a multicentre double-blinded placebo controlled trial performed in a high-risk population of women with a previous spontaneous preterm birth <37 weeks gestation.[1]

1.2. Study objectives

The objective of the APRIL study is to assess the effectiveness of low dose aspirin started early in pregnancy as compared to placebo in the prevention of recurrent spontaneous preterm birth.

1.3. Study design

The APRIL study is a multicentre, randomized, double blinded, placebo controlled trial. It is performed within the Dutch Consortium for Healthcare Evaluation and Research in Obstetrics and Gynaecology – NVOG Consortium 2.0 (https://zorgevaluatienederland.nl/associations/8f056ae2-32ab-4b77-a997-7cd703cb36a7).

1.4. Sample size and power

A difference in reduction of recurrent spontaneous preterm birth from 36% to 23% can be detected if 384 patients are recruited (192 in each arm, beta-error 0.2; alpha-error 0.05). To allow for a loss to follow-up of 5%, a total of 406 participants will be recruited (203 in each arm).

1.5. Study population

All women with a history of spontaneous preterm birth of a singleton pregnancy between 22 – 37 weeks of gestation, presenting at the participating hospitals with a singleton pregnancy before 16 weeks of gestation.

1.5.1. Inclusion criteria

In order to be eligible to participate in this study, a subject must meet the following criteria:

- Pregnant women between 8 and 16 weeks gestation
- ≥18 years of age
- History of spontaneous preterm birth
  Spontaneous preterm birth is defined as: birth following spontaneous contractions with intact membranes or birth after preterm rupture of the membranes at a gestational age between 22 and 37 weeks.

1.5.2. Exclusion criteria

A potential subject who meets any of the following criteria will be excluded from participation in this study in case of:

- Another indication for aspirin during pregnancy;
- Indication for use of other anticoagulants during pregnancy;
- Thrombocytopenia or thrombocytopathy;
- History of Indicated PTB for maternal reasons such as preeclampsia or HELLP syndrome;

- History of indicated PTB for fetal reasons such as IUGR
- Major fetal abnormalities in prior preterm birth pregnancy or current pregnancy;
- Multiple pregnancy either in the prior preterm birth pregnancy or current pregnancy.

1.6. Randomisation and masking

Randomisation to either of the treatment arms will be in a ratio of 1 : 1 using random permuted blocks of sizes two and four. An online randomisation module is used (TEN-ALEA; https://nl.tenalea.net/).

1.7. Ethical approval and consent

1.7.1. Regulation statement

The APRIL study was approved by the Research Ethics Committee of the Amsterdam UMC: MEC Amsterdam [2015_332#C20152636] before starting the trial. Local approval needs to be obtained for each site before participating in the trial.

1.7.2. Consent

Written informed consent is obtained from any patient before enrolment.

1.8. Treatment of subjects

1.8.1. Investigational product

For the purpose of this study, subjects will either be treated with one tablet a day of 80 mg aspirin or placebo, preferably taken in the evening. Treatment will be initiated from 8-16 weeks gestation up to 36 weeks of gestation (or birth, whichever comes first).

1.8.2. Use of co-intervention

Other interventions for the prevention of spontaneous preterm birth such as progesterone, cerclage and the Arabin pessary are allowed as co-intervention.

2. Statistical analysis

2.1. Trial profile

The flow diagram of study participants will be displayed using the CONSORT diagram (Figure 1, section 5.1).

2.2. Analysis populations

2.2.1. Intention to treat (ITT) population

The ITT population will consist of all patients who have given consent and have been allocated one of the two treatments, irrespective of treatment received. Randomised women who appear to fail inclusion and exclusion criteria (eligibility violations) during blinded data review, will be excluded from this analysis. This will only be done for criteria that were present at the time of randomisation. Two clinicians will review such cases and where there are discrepancies, a third will be consulted. The following protocol violations will be considered:

- Another indication for aspirin use during pregnancy (present at baseline)
- Indication for use of other anticoagulants during pregnancy (present at baseline)
- Chromosomal or structural fetal anomalies in prior pregnancy ending in preterm birth
- Chromosomal or major structural fetal anomalies in current pregnancy discovered after inclusion Two members of the research team will decide during blinded data review whether the anomaly is significant and warrants exclusion from further analyses.
- Multiple pregnancy in prior pregnancy ending in preterm birth
- Multiple pregnancy in current pregnancy discovered after inclusion
- Other protocol violations (identified during blinded data reviews)

2.2.2. Per protocol population

A per-protocol analysis will be done as a sensitivity analysis to further investigate the efficacy of treatment. This will only be done for the primary outcome. Protocol violations as mentioned in section 2.2.1. will be excluded from this analysis.

The per-protocol population will be defined as:

- Low-dose aspirin arm: women with medication compliance ≥80%
- Placebo arm: women with medication compliance ≥80%

The definition of treatment compliance can be found in section 2.7.1.

2.3. Baseline characteristics

We will include all baseline characteristics of all participants who were randomized, regardless of whether there was loss to follow-up. Baseline characteristics will be presented as numbers and percentages in each treatment group (low-dose aspirin vs. placebo), or as averages (mean or median) with standard deviations or interquartile ranges as appropriate. A mock table is provided as Table 1 in section 5.2.

2.4. Primary outcome

The primary outcome measure will be preterm birth, defined as birth from 16^{+0} to 36^{+6} weeks gestation. The mock table is presented in section 5.3.1 and the Kaplan-Meier curve in section 5.3.2.

2.5. Secondary outcomes

Where possible, we will assess the core-outcomes for research concerning interventions to prevent preterm birth as defined by the CROWN-initiative.[2] The mock tables are presented in sections 5.3.1, 5.3.3. and 5.3.4.

2.5.1. Neonatal outcomes

<u>Composite poor neonatal outcome:</u>

- Bronchopulmonary dysplasia (BPD)
- Periventricular leukomalacia (PVL) > grade 1
- Intraventricular haemorrhage (IVH) > grade 2
- Necrotizing enterocolitis (NEC) > stage 1
- Retinopathy of premature (ROP)
- Culture proven sepsis
- Mortality

The individual components of the composite perinatal outcome will also be assessed separately. If there are components of the outcome missing, but another component has occurred, then the composite outcome will be considered to have occurred. If many components are missing, but the infant was born >32^{+0} weeks gestation and was not admitted to the NICU, the composite can be considered not to have occurred.

The neonatal outcomes can be found in mock table 3 in section 5.3.3. The definitions are reported section 7.2.

2.5.2. Birth outcomes

- Gestational age at birth
- PPROM <37 weeks gestation
- Preterm birth will be divided into categories according to gestational age. These groups will also be divided according to the onset of preterm birth (spontaneous or indicated).
- Mode of delivery
- Birthweight
- Small for gestational age (birth weight <10th percentile)

The birth outcomes can be found in mock table 2 in section 5.3.1. The definitions are reported in section 7.1.

2.5.3. Maternal outcomes

Maternal outcomes include the interventions received during pregnancy, hospital admissions, maternal self-reported symptoms, interventions during pregnancy, other morbidities and mortality. See table 4 in section 5.3.4. for the mock table of maternal outcomes and see section 7.3 for their definitions.

2.5.4. Additional outcomes in supplementary information

Additional neonatal and maternal outcomes are included in the supplementary tables.
See the mock tables and figure in section 6. The definitions can be found in section 7.1 to 7.3.

2.6. Subgroup analyses

To determine whether a reduced or improved response to aspirin can be predicted, subgroups of the ITT population will be formed according to the following factors:

- Women with a previous preterm birth:
    - before 30 weeks gestational age
    - between 30 and 34 weeks gestational age
    - after 34 weeks gestational age

  In case a woman had multiple spontaneous preterm births, the woman will be classified according to the earliest preterm birth.
- Onset of previous preterm birth: spontaneous contractions with intact membranes versus preterm premature rupture of membranes (PPROM). In case a woman had multiple spontaneous preterm births, the woman will be classified according to the earliest preterm birth.
- Women treated with progestagens versus no additional treatment
- Women with a short cervix (< 25 mm) in the current pregnancy versus longer ≥25 mm

  Based on the shortest cervical length measured during routine cervical length screening in asymptomatic women from 14^{+0} to 23^{+6} weeks.
- Initiation of treatment at 8-12 weeks versus 12-16 weeks gestation

2.7. Safety outcomes

2.7.1. Treatment compliance

The only sensitivity analysis performed will be the per protocol analysis described above in section 2.2.2. This analysis will be based on treatment compliance. It is presented in table 2, section 5.3.1.

After randomisation, the study medication is delivered to the woman's home address. Study medication should be initiated between 8^{+0} and 16^{+0} weeks gestational age. One dose of study medication should be taken daily until 36^{+0} weeks gestation or the start of labour, whichever comes first. For calculation of the expected doses per patient, we will use the patient reported gestational age of therapy initiation. In case reported initiation is later than 16^{+0} gestational age, than 16^{+0} will be used for calculation.

If the gestational age at initiation of therapy is unknown, it will be estimated based on randomisation date. On average, medication delivery is five days after randomisation.

The expected medication use will be calculated as follows:

**Expected doses per patient**	=	**35^{+6} GA**<br>*or*<br>**GA in case of preterm birth**<br>*(whichever comes first)*	–	**GA start medication (max 16^{+0})**<br>*or, if unknown,*<br>**GA at medication delivery***

** Gestational age at randomisation + 5 days*

Women are supplied with diaries to record on which days they took the study medication. In addition, women are required to return all leftover pills after completion or cessation of therapy. The diaries and leftover medication can be used to make an estimate of study medication used by each woman.

In case of discrepancies between the used medication between the diary and the leftover medication, the lowest number of use, leading to the lowest compliance rate, will be used.

Compliance will be calculated as follows:

$$\textbf{Compliance \%} = \frac{\textbf{Number of doses of study medication used}}{\textbf{Expected number of doses for each patient}}$$

Women will be considered compliant when they take the medication ≥80% of the days they should have taken it. Women who did not return their medication diary or leftover study medication have unknown medication adherence and will be excluded from this per protocol analysis.

Treatment compliance will be summarised for all women and separately for both treatment groups.

2.7.2. Premature discontinuation of study medication

Premature discontinuation of study medication is defined as patients who discontinue treatment before 36 weeks gestation for other reasons than preterm birth. The following data on premature discontinuation of study medication will be summarised according to treatment groups in the Supplementary information. See section 6.3.

- Number of women who stopped treatment
- Main reason for premature discontinuation of treatment
    - Women's preference not to continue
    - Investigator terminated participation
    - Vaginal bleeding
    - Side-effects
    - Serious adverse event
    - Episode of threatened preterm labour which did not result in preterm birth
    - Detection of significant structural or chromosomal anomalies after randomisation
    - Other

2.7.3. Adverse events

All serious adverse events (SAE's) occurring during the study will be listed individually in the supplementary information (see section 6.4). Listings will be divided according to treatment group. All serious adverse events that are considered to be possibly related to the study medication by the investigators will be marked.

2.8. Statistical analyses

For dichotomous outcomes, generalized linear regression (glm) analysis using a log link will be performed to calculate Relative Risks. The 95% confidence intervals and the p-value will also be presented. When there are ≤5 events for a variable, Fisher's exact test will be used to calculate the p-value instead of the glm model.
For the primary outcome, the absolute risk difference will be presented. If the relative risk is <1 the number need to treat will be calculated. If the relative risk is >1, number needed to harm will not be calculated. Additionally, Bayes factor analysis will be conducted using the assumptions given in the sample size. A result <1.0 supports the conclusion that aspirin reduces the risk of preterm birth, while a Bayes factor >1.0 supports the inverse conclusion. The suggested threshold in the literature is 0.1 for

Bayes factor as an indicator of a high probability of an intervention effect similar to or even greater than the hypothetical intervention effect used in the sample size calculation.

The distribution of continuous outcomes will be inspected visually. For approximately normally distributed continuous outcomes, means and standard deviations will be reported and difference in means will be calculated. Mean differences and the corresponding 95% confidence intervals will be presented along with the p-value from the t-test. For highly skewed continuous outcomes, medians and interquartile ranges will be reported together with the differences in medians. The p-value from the Mann Whitney U test will also be reported. For outcomes with multiple categories, a chi-square test will be performed.

Subgroup analyses will be conducted by adding an interaction term to the model and testing for the statistical significance of this interaction term. The results of the subgroup analyses will we presented regardless of the statistical significance (in the appendix). For the subgroup analysis on gestational age at prior preterm birth, the subgroup with most participants will be taken as the reference category.

Sensitivity analyses (i.e. per protocol analysis) will be undertaken to test the robustness of conclusions.

The time to preterm birth (<37 weeks) will be illustrated in a Kaplan Meier plot. The X-axis of this plot will be gestational age in weeks and the Y-axis the occurrence of preterm birth. The difference in gestational age at birth between the study arms will be tested with the log-rank test. The number with the event over the number at risk for each time period of 2 weeks will be presented under the chart. A cox-proportional hazards model will also be used when the assumptions of this model have been met (i.e. curves are not overlapping).

All statistical tests will use a 2-sided p-value of 0.05. All confidence intervals presented will be 95% and two-sided. There will be no adjustment of p-values, as no interim analysis was performed. Note: the data was only observed for safety and there were no stopping rules based on the statistical significance of the effect of the treatment.

2.9. Data management and reproducibility of the analyses

All analyses on the primary outcome will be carried out by two researchers (AL and an epidemiologist from the trial bureau). The needed variables for the outcomes will be specified by one researcher to reduce initial discrepancies. Where there are discrepancies, a third researcher will be consulted. No changes to the database will be made after the database has been locked, except through syntax to ensure reproducibility of the results.
Self-reported medication compliance and self-reported symptoms (scale 0-5) were entered in an excel file manually and linked to the data from the electronic Case Report Forms.

2.10. Missing data

Data on the primary outcome (preterm birth <37 weeks gestation) must be collected after birth. Although little dropout is expected a 5% loss to follow-up has been allowed for in the sample size calculation. If loss to follow-up is indeed <5%, no imputation for the primary outcome will be used. If loss to follow up is ≥5%, characteristics of complete and incomplete cases will be presented in an appendix and multiple imputation (n≥10) will be conducted for the primary outcome as a sensitivity analysis. The analysis will be performed on the imputed datasets will be combined using Rubin's rules. This will only be done for the primary intent to treat analysis.

For the baseline table, the denominator will only be presented when the outcome is missing. Otherwise the total number in that group will be assumed to be the denominator.

For the outcome tables, the number of mother-infant pairs randomized to a treatment arm will be considered the denominator. Only when an outcome is missing, does the denominator change. In this case, the smaller denominator will be shown. The calculation of relative risks will exclude participants with missing outcomes. For example, if there is a stillbirth, there is no NICU admission, but NICU admission will not be considered missing, simply absent, and the denominator will remain the same. The same will apply in case of neonatal death and a certain outcome, such as BPD, could not yet have occurred. However, if there is a livebirth, and the variable on NICU admission is absent for one participant, this participant will also be removed from the denominator.

2.11. Interim Analyses and Data Monitoring

No interim analysis for efficacy was planned. A safety analysis was performed after the 98th and 200th inclusion by an independent statistician. The report was reviewed by the data safety monitoring committee. There were no concerns regarding safety and after both reviews the committee advised to continue the trial according to protocol.
At the time of the second safety review, including 200 women with completed follow-up, the total sample size was almost reached. Therefore, the Data Safety Monitoring Committee concluded no additional interim analysis were needed.

Because no analyses on efficacy are performed, no adjustments to the final p-value for testing at the end of the study are necessary.

4. Comparison to study protocol

The current analysis plan is largely based on the published APRIL study protocol.[1] Slight changes were made and these are listed below:

- The study protocol states that the independent Data Safety Monitoring Committee would monitor the patient safety every 6 months, starting 9 months after the inclusion of the first patient. However, due to a rather slow inclusion rate the first months of the trial, the committee decided to perform monitoring every 100 inclusions (with completed follow-up).

- Percentiles of birth weight will now be calculated based on the birthweight chart by Hoftiezer et al. This birth weight chart was not yet available at the time of the production and publication of the study protocol. However, the birth weight chart by Hoftiezer et al is currently most accepted for the calculation of growth centiles in Dutch practice.[3]

- In the published APRIL study protocol we described to analyse preterm birth rates ≤ 28 weeks, ≤ 32 weeks and ≤ 34 weeks gestation. During the establishment of this analysis plan the research team decided to limit the analyses to ≤ 28 and ≤ 34 weeks gestation to reduce multiple testing. Preterm births ≤ 28 weeks, ≤ 34 weeks and ≤ 37 weeks will be analysed as a group and also separately based on the onset of birth: spontaneous or indicated.

5. Mock tables and figures

5.1. Flow diagram of participants

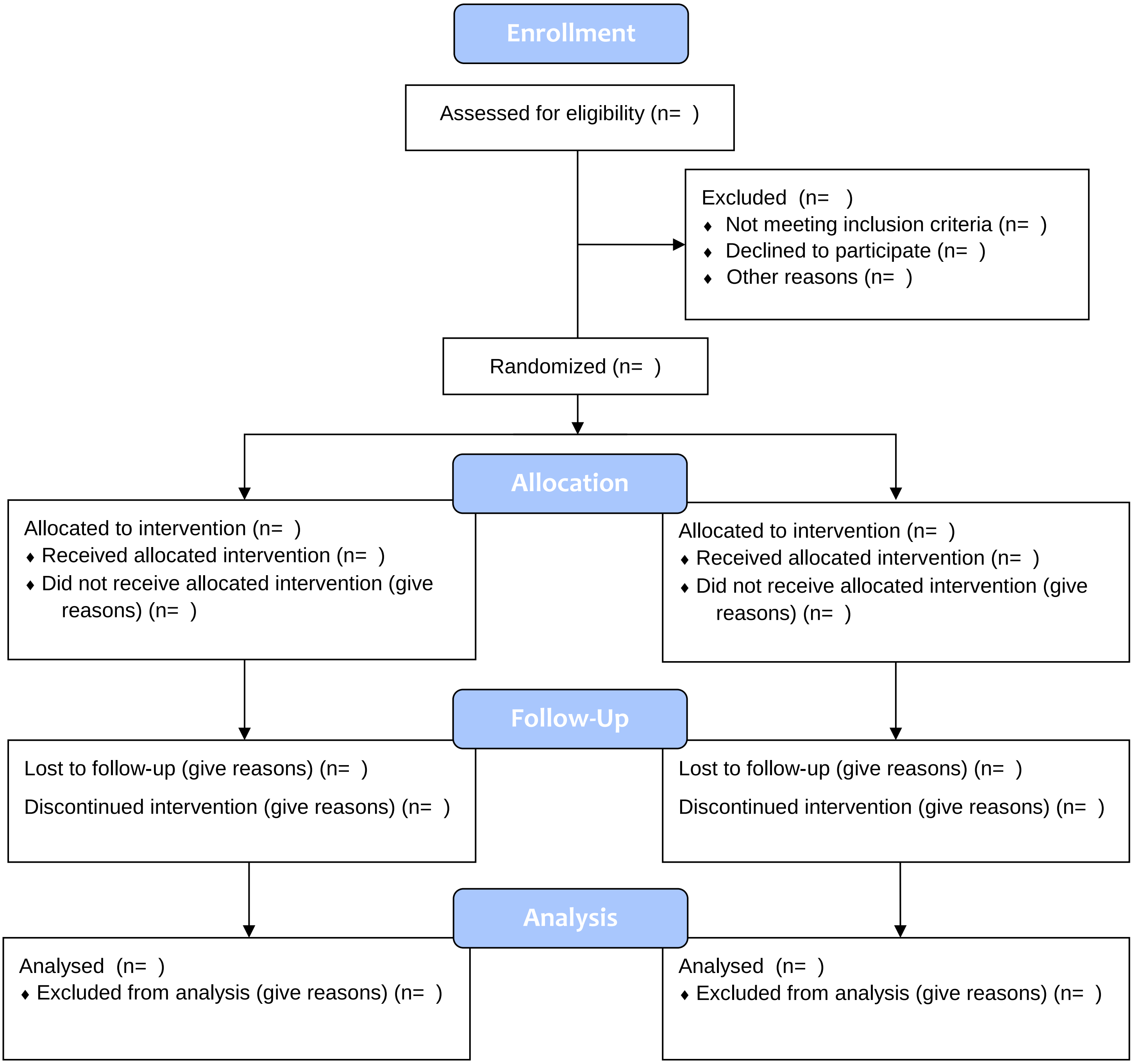

Figure 1 – Flow diagram of participants

5.2. Baseline characteristics

	Aspirin group<br>*(n=NNN)*	Placebo group<br>*(n=NNN)*
**Age – years**	mean (SD) or median [IQR]	mean (SD) or median [IQR]
**Body-mass index (kg/m²)**	mean (SD) or median [IQR]	mean (SD) or median [IQR]
**Ethnic origin**		
White	NNN (%)	NNN (%)
Other origins	NNN (%)	NNN (%)
**Education**		
Low	NNN (%)	NNN (%)
Middle and high	NNN (%)	NNN (%)
**Smoking**		
Yes	NNN (%)	NNN (%)
No	NNN (%)	NNN (%)
Quit	NNN (%)	NNN (%)
**Alcohol**		
Yes	NNN (%)	NNN (%)
No	NNN (%)	NNN (%)
**Method of conception**		
Natural	NNN (%)	NNN (%)
IUI and/or ovulation induction	NNN (%)	NNN (%)
IVF/ICSI	NNN (%)	NNN (%)
**Maternal medical history**		
Diabetes mellitus	NNN (%)	NNN (%)
Gestational diabetes	NNN (%)	NNN (%)
Renal disease	NNN (%)	NNN (%)
Inflammatory Bowel Disease	NNN (%)	NNN (%)
Thyroid disease	NNN (%)	NNN (%)
Chronic hypertension	NNN (%)	NNN (%)
Systemic Lupus Erythematosus	NNN (%)	NNN (%)
Cardiac disease	NNN (%)	NNN (%)
**Obstetric history**		
Parity		
One	NNN (%)	NNN (%)
Two	NNN (%)	NNN (%)
≥Three	NNN (%)	NNN (%)
Number of previous spontaneous preterm births (22^{+0}-37^{+0})		
One	NNN (%)	NNN (%)
≥Two	NNN (%)	NNN (%)
Number of midtrimester fetal loss (16^{+0} -21^{+6})		
One	NNN (%)	NNN (%)
≥Two	NNN (%)	NNN (%)
Number of therapeutic abortions		
One	NNN (%)	NNN (%)
≥Two	NNN (%)	NNN (%)
Number of miscarriages and ectopic pregnancies (<16^{+0})		
One	NNN (%)	NNN (%)
≥Two	NNN (%)	NNN (%)
**Risk factors for preterm birth**		

History of cervical surgery (conisation/LLETZ)	NNN (%)	NNN (%)
History of uterine surgery (e.g. myomectomy)	NNN (%)	NNN (%)
Cerclage in previous pregnancy	NNN (%)	NNN (%)
Uterus anomaly	NNN (%)	NNN (%)
Family history (mother/sister) of preterm birth	NNN (%)	NNN (%)
Short interpregnancy interval (<6 months from last pregnancy to conception)	NNN (%)	NNN (%)
**Gestational age at randomisation** – weeks	mean (SD) or median [IQR]	mean (SD) or median [IQR]
**Fetal gender** – girl	NNN (%)	NNN (%)

Table 1 – Baseline characteristics of all randomized participants

5.3. Primary and secondary outcomes

5.3.1. Birth outcomes

	Aspirin group *(n=NNN)*	Placebo group *(n=NNN)*	Relative Risk (95% CI)	p-value
**Preterm birth at <37 weeks gestation** (ITT analysis)	NNN (%)	NNN (%)	RR (95% CI)	0.XX
Spontaneous onset of birth	NNN (%)	NNN (%)	RR (95% CI)	0.XX
Indicated	NNN (%)	NNN (%)	RR (95% CI)	0.XX
**Preterm birth <37 weeks gestation** (per protocol analysis)*	NNN (%)	NNN (%)	RR (95% CI)	0.XX
**Prelabour rupture of membranes** (<37 weeks gestation)	NNN (%)	NNN (%)	RR (95% CI)	0.XX
**Gestational age at birth (weeks + days)**	mean (SD) or median [IQR]	mean (SD) or median [IQR]	RR (95% CI)	0.XX
**Time between randomisation and birth (weeks + days)**	mean (SD) or median [IQR]	mean (SD) or median [IQR]	RR (95% CI)	0.XX
**Preterm birth ≤34 weeks gestation**	NNN (%)	NNN (%)	RR (95% CI)	0.XX
Spontaneous onset of birth	NNN (%)	NNN (%)	RR (95% CI)	0.XX
Indicated	NNN (%)	NNN (%)	RR (95% CI)	0.XX
**Preterm birth ≤28 weeks gestation**	NNN (%)	NNN (%)	RR (95% CI)	0.XX
Spontaneous onset of birth	NNN (%)	NNN (%)	RR (95% CI)	0.XX
Indicated	NNN (%)	NNN (%)	RR (95% CI)	0.XX
**Midtrimester fetal loss (16^{+0} -21^{+6})**	NNN (%)	NNN (%)	RR (95% CI)	0.XX
**Mode of birth**				
Spontaneous vaginal birth	NNN (%)	NNN (%)	RR (95% CI)	0.XX
Assisted vaginal birth	NNN (%)	NNN (%)	RR (95% CI)	0.XX
Caesarean delivery	NNN (%)	NNN (%)	RR (95% CI)	0.XX
**Postpartum haemorrhage**				
>500 mL	NNN (%)	NNN (%)	RR (95% CI)	0.XX
>1000 mL	NNN (%)	NNN (%)	RR (95% CI)	0.XX
**Birthweight (g)**	mean (SD) or median [IQR]	mean (SD) or median [IQR]	RR (95% CI)	0.XX
**Small for gestational age** (<10th percentile)	NNN (%)	NNN (%)	RR (95% CI)	0.XX

Table 2 – Birth outcomes

* The per protocol analysis only includes women with ≥80% compliance to therapy

5.3.2. Kaplan-Meier plot

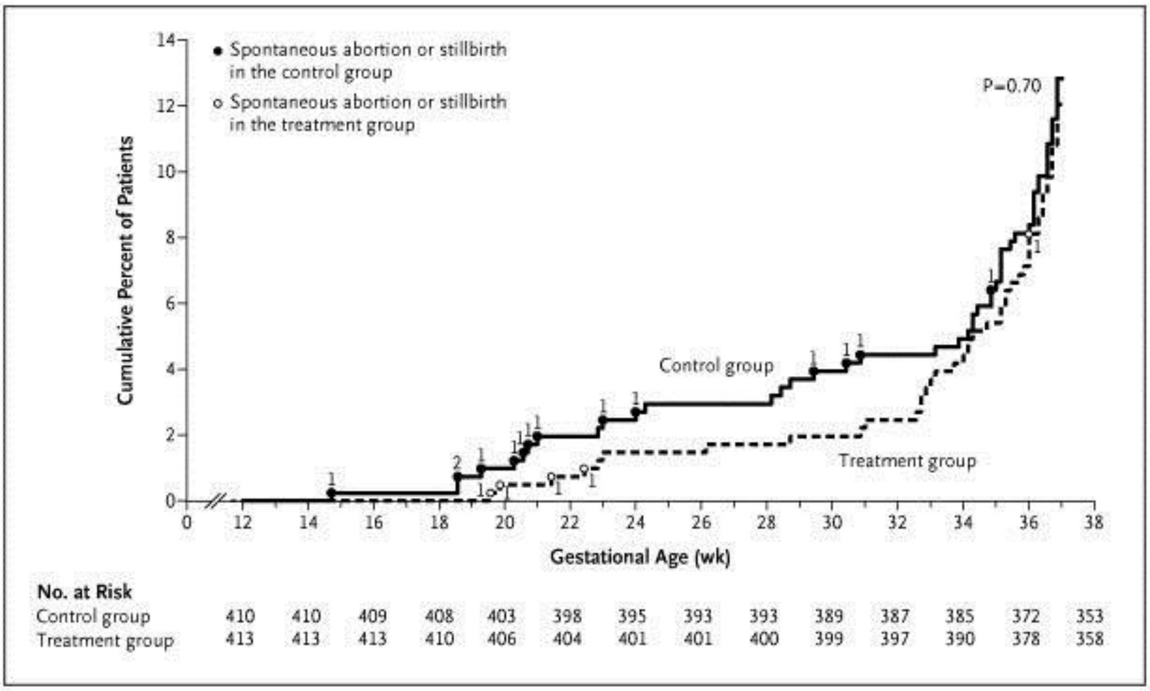

Figure 2 – Kaplan-Meier Curve for the cumulative incidence of preterm birth at 16 to 37 weeks gestation

Following example from Michalowicz et al. NEJM 2006[4]
Y-axis: Incidence preterm birth <37 weeks gestation (%)
X-axis: Gestational age in weeks
Special markings for spontaneous abortions and fetal death

5.3.3. Neonatal outcomes

	Aspirin group<br>*(n=XXX)*	Placebo group<br>*(n=XXX)*	Relative Risk (95% CI)	p-value
**Composite poor neonatal outcome**	NNN (%)	NNN (%)	RR (95% CI)	0.XX
BPD	NNN (%)	NNN (%)	RR (95% CI)	0.XX
PVL > grade 1	NNN (%)	NNN (%)	RR (95% CI)	0.XX
IVH > grade 2	NNN (%)	NNN (%)	RR (95% CI)	0.XX
NEC > stage 1	NNN (%)	NNN (%)	RR (95% CI)	0.XX
Retinopathy of prematurity	NNN (%)	NNN (%)	RR (95% CI)	0.XX
Culture proven sepsis	NNN (%)	NNN (%)	RR (95% CI)	0.XX
Mortality	NNN (%)	NNN (%)	RR (95% CI)	0.XX
**Mortality**				
Fetal death	NNN (%)	NNN (%)	RR (95% CI)	0.XX
Neonatal death	NNN (%)	NNN (%)	RR (95% CI)	0.XX
**Days of admission on the NICU until 3 months corrected age**	mean (SD) or median [IQR]	mean (SD) or median [IQR]	RR (95% CI)	0.XX
**Total days in hospital until 3 months corrected age**	mean (SD) or median [IQR]	mean (SD) or median [IQR]	RR (95% CI)	0.XX

Table 3 – Neonatal outcomes

5.3.4. Maternal outcomes

	Aspirin group (n=XXX)	Placebo group (n=XXX)	Relative Risk (95% CI)	p-value
**Maternal mortality**	NNN (%)	NNN (%)	RR (95% CI)	0.XX
**Maternal morbidity**				
Gestational diabetes	NNN (%)	NNN (%)	RR (95% CI)	0.XX
Pregnancy-induced hypertension	NNN (%)	NNN (%)	RR (95% CI)	0.XX
Preeclampsia/HELLP syndrome	NNN (%)	NNN (%)	RR (95% CI)	0.XX
Eclampsia	NNN (%)	NNN (%)	RR (95% CI)	0.XX
Pulmonary oedema	NNN (%)	NNN (%)	RR (95% CI)	0.XX
Thromboembolic disease	NNN (%)	NNN (%)	RR (95% CI)	0.XX
Placental abruption	NNN (%)	NNN (%)	RR (95% CI)	0.XX
**Maternal self-reported symptoms (moderate to severe)**				
Vaginal bleeding	NNN (%)	NNN (%)	RR (95% CI)	0.XX
Other bleeding	NNN (%)	NNN (%)	RR (95% CI)	0.XX
Gastro-intestinal complaints	NNN (%)	NNN (%)	RR (95% CI)	0.XX
**Women admitted for vaginal bleeding during pregnancy**	NNN (%)	NNN (%)	RR (95% CI)	0.XX
**Women admitted for threatened preterm labour**	NNN (%)	NNN (%)	RR (95% CI)	0.XX
**Course of steroids for fetal lung maturity**	NNN (%)	NNN (%)	RR (95% CI)	0.XX
**Tocolytic therapy**	NNN (%)	NNN (%)	RR (95% CI)	0.XX
**Hospital admission during pregnancy for any reason (days)**	mean (SD) or median [IQR]	mean (SD) or median [IQR]	RR (95% CI)	0.XX
**Interventions during pregnancy**				
Progesterone treatment	NNN (%)	NNN (%)	RR (95% CI)	0.XX
Cerclage placement	NNN (%)	NNN (%)	RR (95% CI)	0.XX
Pessary placement	NNN (%)	NNN (%)	RR (95% CI)	0.XX
Bacterial vaginosis treated with antibiotics	NNN (%)	NNN (%)	RR (95% CI)	0.XX
**Urinary tract or genital infections treated with antibiotics**	NNN (%)	NNN (%)	RR (95% CI)	0.XX

Table 4 – Interventions during pregnancy, hospital admissions and maternal outcomes

5.4. Subgroup analyses

	PTB < 37 weeks in aspirin group (n=NNN)	PTB < 37 weeks in placebo group (n=NNN)	Relative Risk (95% CI)	p-value subgroup	p-value interaction term
**Initiation of treatment**					
8^{+0} to 11^{+6} weeks gestation	NNN (%)	NNN (%)	RR (95% CI)	0.XX	0.XX
12^{+0} to 16^{+0} weeks gestation	NNN (%)	NNN (%)	RR (95% CI)	0.XX	
**Progesterone treatment during pregnancy**					
Progesterone	NNN (%)	NNN (%)	RR (95% CI)	0.XX	0.XX
No progesterone	NNN (%)	NNN (%)	RR (95% CI)	0.XX	
**Cervical length in the current pregnancy (14^{+0} - 23^{+6} weeks gestation)**					
< 25 mm	NNN (%)	NNN (%)	RR (95% CI)	0.XX	0.XX
≥ 25 mm	NNN (%)	NNN (%)	RR (95% CI)	0.XX	
**Women with a previous preterm birth**					
< 30^{+0} weeks gestation	NNN (%)	NNN (%)	RR (95% CI)	0.XX	0.XX
30^{+0} - 33^{+6} weeks gestation	NNN (%)	NNN (%)	RR (95% CI)	0.XX	0.XX
34^{+0} - 36^{+6} weeks gestation	NNN (%)	NNN (%)	RR (95% CI)	0.XX	0.XX
**Onset of previous spontaneous preterm birth**					
Spontaneous contractions with intact membranes	NNN (%)	NNN (%)	RR (95% CI)	0.XX	0.XX
Preterm prelabour rupture of membranes	NNN (%)	NNN (%)	RR (95% CI)	0.XX	

Table 6 – Subgroup analyses on the primary outcome

6. Supplementary tables

6.1. Additional neonatal outcomes

	**Aspirin group** *(n=XXX)*	**Placebo group** *(n=XXX)*
Number of days on ventilation support (intubation + CPAP)	mean (SD)	mean (SD)
IRDS requiring surfactant therapy	NNN (%)	NNN (%)
Patent ductus arteriosus (PDA) requiring treatment	NNN (%)	NNN (%)
Hypotension requiring inotropics	NNN (%)	NNN (%)
Intestinal perforation	NNN (%)	NNN (%)
Convulsions	NNN (%)	NNN (%)
Asphyxia	NNN (%)	NNN (%)
Pneumothorax/pneumomediastinum	NNN (%)	NNN (%)
Early onset neonatal sepsis, culture proven	NNN (%)	NNN (%)
Late onset neonatal sepsis, culture proven	NNN (%)	NNN (%)
Neonatal meningitis	NNN (%)	NNN (%)
Cerebellar bleeding	NNN (%)	NNN (%)
Cephalic hematoma	NNN (%)	NNN (%)
Other cerebral bleeding	NNN (%)	NNN (%)
Gastro intestinal bleeding	NNN (%)	NNN (%)
Lung bleeding	NNN (%)	NNN (%)
Other bleeding	NNN (%)	NNN (%)
Congenital anomalies & genetic disorders	NNN (%)	NNN (%)

Supplementary table 1 – Additional neonatal outcome

6.2. Additional maternal outcomes

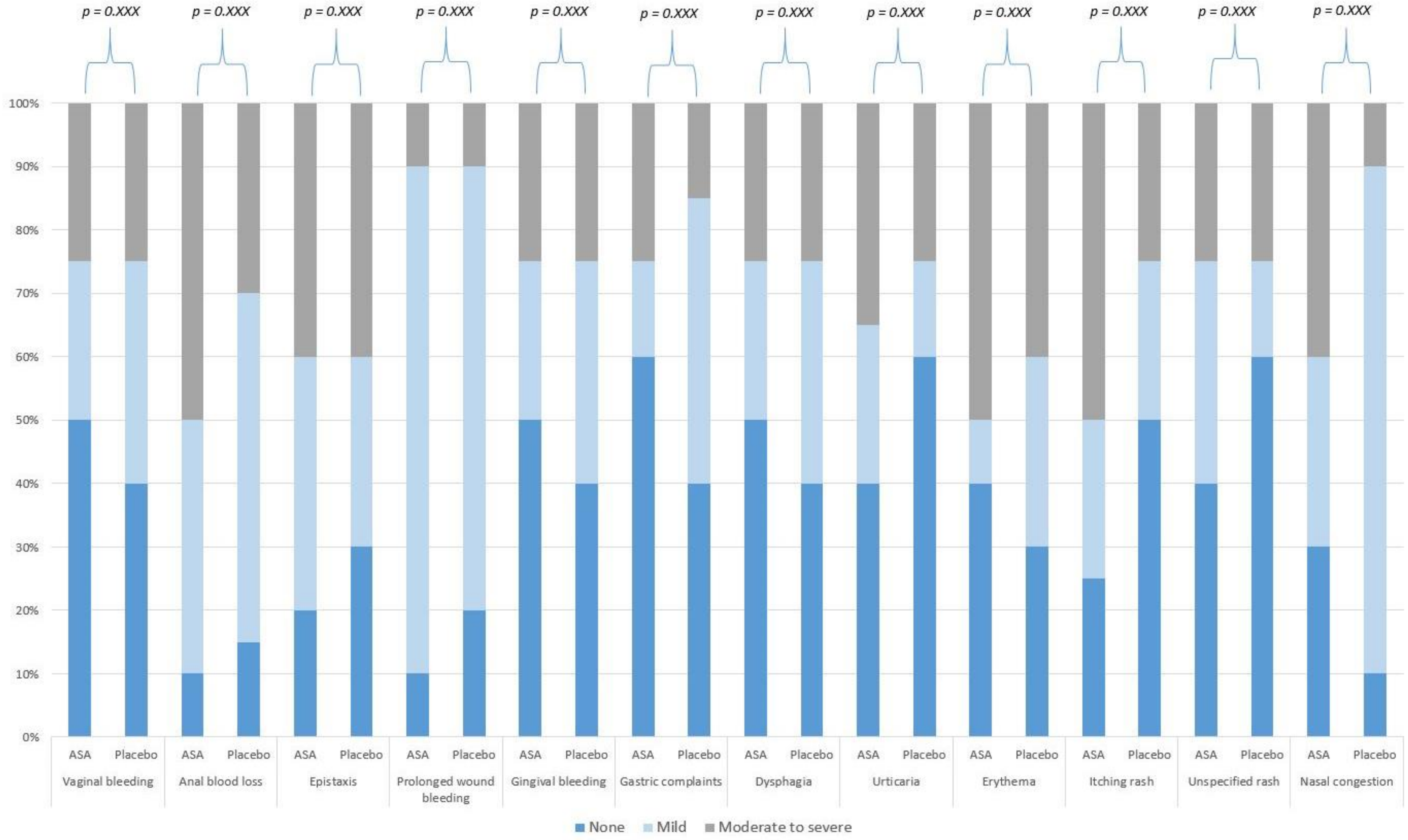

Data presented in the bar charts is fictional for the purpose of illustration.

Supplementary figure 1 – Self-reported maternal symptoms: none, mild or moderate to severe

6.3. Reasons for premature discontinuation of study medication

	Aspirin group<br>*(n=XXX)*	Placebo group<br>*(n=XXX)*
**Medication discontinued**	NNN (%)	NNN (%)
**Main reasons for discontinuation**		
Women's preference	NNN (%)	NNN (%)
Vaginal bleeding	NNN (%)	NNN (%)
Side-effects	NNN (%)	NNN (%)
Serious adverse event	NNN (%)	NNN (%)
Episode of threatened preterm labour which did not result in preterm birth	NNN (%)	NNN (%)
Detection of significant structural or chromosomal anomalies after randomisation	NNN (%)	NNN (%)
Investigator terminated participation	NNN (%)	NNN (%)

Supplementary table 3 – Reasons for discontinuation of study medication according to treatment group

6.4. List of Serious Adverse Events (SAEs)

**Description of event**	**Aspirin group (n=XXX)**	**Placebo group (n=XXX)**
**Maternal**		
…	NNN	NNN
…	NNN	NNN
**Fetal chromosomal abnormalities**		
…	NNN	NNN
…	NNN	NNN
**Fetal structural defects**		
…	NNN	NNN
…	NNN	NNN
**At least one serious adverse event, no. (%)**	NNN (%)	NNN (%)
**No serious adverse event, no (%)**	NNN (%)	NNN (%)

Supplementary table 4 – List of Serious Adverse Events (SAEs)

All serious adverse events marked with "*" were considered to be possibly related to the intervention by the investigators.

6.5. Number of inclusions per participating center

Centre	Acetylsalicylic acid<br><br>N=NNN	Placebo<br><br>N=NNN	Total<br><br>N=NNN
Amsterdam UMC, location AMC	NN	NN	NN
Amsterdam UMC, location VUmc	NN	NN	NN
Amphia Ziekenhuis	NN	NN	NN
Bethesda Ziekenhuis	NN	NN	NN
Deventer ziekenhuis	NN	NN	NN
Diakonessenhuis	NN	NN	NN
Flevoziekenhuis	NN	NN	NN
Gelre Ziekenhuis Apeldoorn	NN	NN	NN
Haga Ziekenhuis	NN	NN	NN
Jeroen Bosch Ziekenhuis	NN	NN	NN
Leiden Universitair Medisch Centrum	NN	NN	NN
Maastricht Universitair Medisch Centrum	NN	NN	NN
Martini ziekenhuis	NN	NN	NN
Maxima Medisch Centrum	NN	NN	NN
Medisch Centrum Haaglanden	NN	NN	NN
Ommelander Ziekenhuis	NN	NN	NN
Onze Lieve Vrouwe Gasthuis Oost	NN	NN	NN
Reinier de Graaf Groep	NN	NN	NN
Spaarne Ziekenhuis	NN	NN	NN
St Antonius ziekenhuis	NN	NN	NN
Tergooi Ziekenhuizen	NN	NN	NN
Universitair Medisch Centrum Groningen	NN	NN	NN
Universitair Medisch Centrum St. Radboud	NN	NN	NN
Universitair Medisch Centrum Utrecht	NN	NN	NN
Wilhelmina Ziekenhuis	NN	NN	NN
Ziekenhuisgroep Twente	NN	NN	NN
Zuyderland Ziekenhuis	NN	NN	NN

Supplementary table 5 – Number of inclusions per centre

7. Definitions of outcomes

7.1. Definitions of birth outcomes

Outcome	Definition
**Preterm birth**	Birth from 16^{+0} to 36^{+6} weeks gestation
Spontaneous onset	Preterm birth following either the spontaneous onset of contractions with intact membranes or prelabour rupture of membranes
Indicated	Medically induced labour or a primary caesarean delivery because of maternal or fetal complication
**Midtrimester fetal loss**	Fetal loss from 16^{+0} to 21^{+6} weeks gestation after spontaneous contractions or spontaneous rupture of the membranes.
**Small for gestational age**	Birthweight <10th percentile for the gestational age using the birthweight chart by Hoftiezer et al.[3]

7.2. Definitions of neonatal outcomes

Outcome	Definition
**Asphyxia**	This is diagnosed in case of an Apgar score after 5 minutes ≤5 or resuscitation/ventilation for 10 minutes after birth or pH<7.0 and base excess >16 mmol/L (fetal blood taken form the umbilical cord).[5]
**BPD**	Infants receiving supplemental oxygen for at least 28 days at 36 weeks postmenstrual age. Classification will take place using an oxygenreductiontest.[6]
**Culture proven sepsis**	This is diagnosed by the combination of clinical signs of infection and positive blood cultures. Distinction will be made between:
Early onset sepsis (EONS)	clinical suspicion and positive blood culture <72 hours
Late onset sepsis (LONS)	clinical suspicion, positive blood culture and a CRP blood level of >10mg/L >72 hours[7]
**IVH > grade 2**<br>**PVL > grade 1**	This will be diagnosed by repeated neonatal cranial ultrasound by a neonatologist according to the guidelines on neuroimaging of the Papille classification described by de Vries and Ment et al.[8,9]
**NEC > stage 1**	Diagnosis according to Bell staging criteria.[10]
**Pneumothorax**	Visible on chest x-ray and/or the need to perform drainage.
**Proven meningitis**	Clinical suspicion of meningitis supported by positive liquor culture or liquor analysis highly suggestive for meningitis.
**Retinopathy of prematurity**	grading following the international classification of retinopathy of prematurity.[11,12]
**Mortality**	Death of a fetus or neonate at any time between a gestational age ≥16 weeks and discharge.
Fetal death	Death during pregnancy (≥16 weeks) or during labour
Neonatal death	Death occurring in the period after birth until discharge

7.3. Definitions of maternal outcomes

Outcome	Definition
**Eclampsia**	Seizures in a pregnancy complicated by preeclampsia.
**Gestational diabetes**	Onset of diabetes after 20 weeks gestational age.
**HELLP-syndrome**	Combination of haemolysis, elevated liver enzymes and thrombocytopenia with or without the presence of proteinuria or hypertension.
**Hospital admissions for threatened preterm labour**	Number and percentage of women with an admission for threatened preterm labour.
**Hospital admissions for vaginal bleeding**	Number and percentage of women with an admission for vaginal bleeding during pregnancy.
**Hospital admissions for any reason**	Total days admitted to a hospital for any reason
**Maternal infection**	clinical diagnosis of urinary or genital tract infections and treated with antibiotics
**Maternal mortality**	Death up to 28 days post estimated delivery date
**Maternal self-reported symptoms**	Women receive a diary to score the following complaints: vaginal bleeding/spotting, anal blood loss, epistaxis, prolonged wound bleeding, gingival bleeding, gastric complaints, trouble swallowing, urticaria, erythema, itching rash, unspecified rash and nasal congestion. Women could score these complaints from 0-5 and indicate at which gestational age they experienced the complaints. The scores in the diary were defined as follows:<br>0 = none<br>1 = little imposition<br>2 = some imposition<br>3 = imposition<br>4 = a lot of imposition<br>5 = unbearable imposition<br><br>For analysis we redefined the scores to the following categories:<br>No symptoms = 0<br>Mild symptoms = 1+2<br>Moderate to severe = 3+4+5
**Placental abruption**	Clinical diagnosis of (partial) detachment of the placenta from the uterine wall.
**Preeclampsia**	Pregnancy-induced hypertension in combination with proteinuria (≥300 mg in 24 hours) or thrombocytopenia (<150 x10e9/L), impaired liver function or renal insufficiency. This may occur during pregnancy or the postpartum period.[13]
**Pregnancy-induced hypertension**	New onset of hypertension (≥ 140 mmHg systolic and/or ≥90 mmHg diastolic blood pressure) after 20 weeks gestation measured on at least two occasions four hours apart.[13]
**Pulmonary oedema**	Based on clinical findings
**Thromboembolic disease**	Deep vein thrombosis or pulmonary embolism
**Tocolytic therapy**	Received tocolytic therapy during pregnancy for the delay/prevention of spontaneous preterm birth.

References

1. Visser L, de Boer MA, de Groot CJM, et al. Low dose aspirin in the prevention of recurrent spontaneous preterm labour - the APRIL study: a multicenter randomized placebo controlled trial. BMC Pregnancy Childbirth 2017;17:223.
2. van 't Hooft J, Duffy JM, Daly M, et al. A Core Outcome Set for Evaluation of Interventions to Prevent Preterm Birth. Obstet Gynecol 2016;127:49-58.
3. Hoftiezer L, Hof MHP, Dijs-Elsinga J, Hogeveen M, Hukkelhoven C, van Lingen RA. From population reference to national standard: new and improved birthweight charts. Am J Obstet Gynecol 2019;220:383 e1- e17.
4. Michalowicz BS, Hodges JS, DiAngelis AJ, et al. Treatment of periodontal disease and the risk of preterm birth. N Engl J Med 2006;355:1885-94.
5. Cornette L. Therapeutic hypothermia in neonatal asphyxia. Facts Views Vis Obgyn 2012;4:133-9.
6. Jobe AH, Bancalari E. Bronchopulmonary dysplasia. Am J Respir Crit Care Med 2001;163:1723-9.
7. Stoll BJ, Hansen N, Fanaroff AA, et al. Late-onset sepsis in very low birth weight neonates: the experience of the NICHD Neonatal Research Network. Pediatrics 2002;110:285-91.
8. de Vries LS, Liem KD, van Dijk K, et al. Early versus late treatment of posthaemorrhagic ventricular dilatation: results of a retrospective study from five neonatal intensive care units in The Netherlands. Acta Paediatr 2002;91:212-7.
9. Ment LR, Bada HS, Barnes P, et al. Practice parameter: neuroimaging of the neonate: report of the Quality Standards Subcommittee of the American Academy of Neurology and the Practice Committee of the Child Neurology Society. Neurology 2002;58:1726-38.
10. Kliegman RM, Walsh MC. Neonatal necrotizing enterocolitis: pathogenesis, classification, and spectrum of illness. Curr Probl Pediatr 1987;17:213-88.
11. An international classification of retinopathy of prematurity. The Committee for the Classification of Retinopathy of Prematurity. Arch Ophthalmol 1984;102:1130-4.
12. An international classification of retinopathy of prematurity. II. The classification of retinal detachment. The International Committee for the Classification of the Late Stages of Retinopathy of Prematurity. Arch Ophthalmol 1987;105:906-12.
13. American College of O, Gynecologists, Task Force on Hypertension in P. Hypertension in pregnancy. Report of the American College of Obstetricians and Gynecologists' Task Force on Hypertension in Pregnancy. Obstet Gynecol 2013;122:1122-31.
