# Supplementary material for: Evaluation of low-dose aspirin in the prevention of recurrent spontaneous preterm labour (the APRIL study): A multicentre, randomised, double-blinded, placebo-controlled trial
Source: PLoS Med. 2022 Feb 1;19(2):e1003892. doi: 10.1371/journal.pmed.1003892 (PMC8806064; doi:10.1371/journal.pmed.1003892)
Supplement: Attachment — Submitted filename: APRIL Response editors and reviewers PLOS Medicine 20211009.docx [file pmed.1003892.s012.docx]

Amsterdam, 5 October 2021

Dear editors, dear reviewers,

We are grateful to the editors and reviewers for their insightful comments on our paper. We have been able to incorporate changes to reflect most of the provided suggestions. We have marked the changes within the manuscript by using track changes. Here is a point-by-point response to the editor’s and reviewers' comments and concerns. The reported line numbers correspond with the manuscript file including tracked changes.

**COMMENTS FROM THE ACADEMIC EDITOR:**

Well conducted, well written, good supplementary material and limitations clearly presented. Two minor points:

1. Women were excluded if they had another indication for aspirin. What are the indications for aspirin in the Netherlands? Interestingly some trial participants had conditions that would indicate aspirin according to UK/US guidelines (eg chronic hypertension).
   - Response: At the time of study design and the start of the inclusions, there was no Dutch national guideline on the indications for aspirin prophylaxis in pregnancy. In 2018, the Dutch national guideline ([Acetylsalicylzuur pre-eclampsie zwangerschap - Richtlijn - Richtlijnendatabase](https://richtlijnendatabase.nl/richtlijn/hypertensieve_aandoeningen_in_de_zwangerschap/acetylsalicylzuur_pre-eclampsie_zwangerschap.html)) included a recommendation on the prescription of aspirin during pregnancy based on the NICE guidelines. In the APRIL study, screening for study eligibility, e.g. other indications for aspirin use, was left at the discretion of the health care providers in the participating centres. The reason that women with e.g. chronic hypertension were included in the APRIL study, is probably that the screening for risk factors for preeclampsia according to the current NICE guidelines was not fully implemented in all hospitals at that time. We clarified this in the Methods section of the manuscript (line 196-197).
2. Dose could be discussed in the discussion - some studies have advocated 150mg rather than 75 or 80mg.
   - Response: We briefly discuss aspirin dosing in line 443-446. We believe there is insufficient evidence to advocate for higher aspirin doses of 150 mg at this time. Suggestions that aspirin 150 mg might be superior to aspirin 75-80 mg for the prevention of preeclampsia, are based on meta-analyses comparing RCTs with aspirin doses <100 mg to RCTs with aspirin doses ≥100 mg. Other factors could also have contributed to the observed effect in these meta-analyses, such as adherence to medication and population differences. One of the most recent RCTs using aspirin 150 mg for the prevention of preeclampsia (ASPRE trial, Rolnik et al, NEJM), for example, had very high medication adherence rates. In the field of preeclampsia, researchers should perform trials directly comparing aspirin 75-80 mg to 150 mg. For the prevention of (spontaneous) preterm birth, we encourage researchers to perform a large RCT with three arms: placebo, aspirin 75-80 mg, and aspirin 150 mg. We’ve added our recommendation for future research in the final paragraph of the discussion (line 483-493).

**REQUESTS FROM THE EDITORS:**

1. General comments: Please replace the term “compliance” with “adherence” where it is used to refer to treatment adherence.
   - Response: The term “compliance” has been replaced by “adherence” throughout the manuscript.
2. Throughout the paper, please adapt reference call-outs to the following style: "... rate of preterm birth [9,10]." (noting the absence of spaces within the square brackets).
   - Response: We have changed the reference style accordingly throughout the manuscript.

**Data availability:**

PLOS Medicine requires that the de-identified data underlying the specific results in a published article be made available, without restrictions on access, in a public repository or as Supporting Information at the time of article publication, provided it is legal and ethical to do so.  If the data are owned by a third party but freely available upon request, please note this and state the owner of the data set and contact information for data requests (web or email address). Please note that a study author cannot be the contact person for the data. If the data are not freely available, please describe briefly the ethical, legal, or contractual restriction that prevents you from sharing it, and include an appropriate contact (web or email address) for inquiries (again, this cannot be a study author).

- Response: The data are owned by Amsterdam University Medical Centers, location Academic Medical Center (AMC), that supports data sharing. For enquiries on data sharing, a methodologist from the trial centre within the Dutch Consortium for Healthcare Evaluation and Research in Obstetrics and Gynaecology (NVOG Consortium) may be contacted via [trialbureau@zorgevaluatienederland.nl](mailto:trialbureau@zorgevaluatienederland.nl).

**Title:**

Please revise your title according to PLOS Medicine's style. Please place the study design in the subtitle (ie, after a colon). We suggest ‘Evaluation of Aspirin for the Prevention of Recurrent Spontaneous Preterm Labour (the APRIL study): a multicentre, randomised, double-blinded, placebo-controlled trial” or similar.

- Response: The title has been changed to the suggested title above (line 1-3).

**Abstract:**
Please report your abstract according to CONSORT for abstracts, following the PLOS Medicine abstract structure (Background, Methods and Findings, Conclusions)

- Response: The abstract has been edited based on the CONSORT guideline for abstracts and according to the PLOS Medicine abstract structure.

**Abstract Methods and Findings:**

1. Please provide brief demographic details of the study population (e.g. sex, age, ethnicity, etc)
   - Response: Brief demographic details have been added to the abstract (line 101).
2. Line 96 - please define RRR at first use
   - Response: Relative Risk Reduction (RRR) was changed to Relative Risk (RR) and defined at first use in the abstract (line 104).
3. Line 97 - please replace ‘compliant’ with another term
   - Response: We replaced “compliant” with “medication adherence” (line 105).
4. Please state that analysis was intention to treat.
   - Response: We included a description of the intention-to-treat approach in the Methods and Findings section (line 99).
5. Please provide the actual number of events in addition to % and RRR
   - Response: The actual number of events was added to the findings in the abstract (line 105-106 and line 110).
6. Please specify what is meant by ‘poor neonatal outcome’
   - Response: We added the definition of the composite of the poor neonatal outcome to the Methods and Findings section (line 96-99).
7. Please include a summary of adverse events.
   - Response: A brief summary of the serious adverse events was also added to the Methods and Findings section (line 111-113).
8. In the last sentence of the Abstract Methods and Findings section, please describe 2-3 of  the main limitations of the study's methodology.
   - Response: We added a description of the main limitation, which is the underpowered sample size, at the end of the Methods and Findings section (line 113-114).

**Abstract Conclusions:**

Please begin your Abstract Conclusions with "In this study, we observed ..." or similar, to summarize the main findings from your study. Please emphasize what is new and address the implications of your study, being careful to avoid assertions of primacy.

- Response: As suggested, we now begin the Abstract Conclusions with “In this study, we observed…” (line 115).

**Author Summary:**

At this stage, we ask that you include a short, non-technical Author Summary of your research to make findings accessible to a wide audience that includes both scientists and non-scientists. The Author Summary should immediately follow the Abstract in your revised manuscript. This text is subject to editorial change and should be distinct from the scientific abstract. Please see our author guidelines for more information:

In the final bullet point of ‘What Do These Findings Mean?’, please describe the main limitations of the study in non-technical language.

- Response: We have added an Author Summary on Page 6, including the main limitation of the study which is the underpowered sample size.

**Introduction:**
Line 127 - Please temper assertions of primacy by adding ‘to the best of our knowledge’ or similar.

- Response: We added “To the best of our knowledge, … ” to the intended sentence (line 169-170).

**Methods:**
Thank you for providing a CONSORT checklist. Please add the following statement, or similar, to the Methods: "This study is reported as per the Consolidated Standards of Reporting Trials (CONSORT) checklist (S1 Checklist)." When completing the checklist, please use section and paragraph numbers, rather than page numbers which will likely no longer correspond to the appropriate sections after copy-editing.

- Response: We have inserted the suggested sentence in the manuscript (line 187-188) and provided the CONSORT checklist as Appendix S2 by using section and paragraph numbers.

Thank you for providing your prospective protocol and statistical analysis plan. Please make sure that the Methods section transparently describes when analyses were planned, and if/when reported analyses differed from those that were planned. Changes in the analysis-- including those made in response to peer review comments-- should be identified as such in the Methods section of the paper, with rationale. If a reported analysis was performed based on an interesting but unanticipated pattern in the data, please be clear that the analysis was data-driven.

- Response: Analyses were planned before deblinding of treatment allocation and statistical analysis of the results. No changes have been made in the analysis (line 270), nor are changes necessary following peer review comments (only clarifications of the used methodology). Furthermore, all reported analyses were pre-specified and not data-driven.

**Results**
Please incorporate Fig S1 into the main paper.

- Response: Figure S1, the Kaplan-Meier Curve, has now been incorporated as Figure 2 in the main paper (line 355, 365).

**Discussion:**

1. Please present and organize the Discussion as follows: a short, clear summary of the article's findings; what the study adds to existing research and where and why the results may differ from previous research; strengths and limitations of the study; implications and next steps for research, clinical practice, and/or public policy; one-paragraph conclusion.
   - Response: We have rearranged the Discussion as suggested by the editor (line 425-482). Furthermore, we have added an paragraph including suggested next steps for research (line 483-493).
2. Please remove all subheadings within your Discussion e.g. Strengths and limitations
   - Response: We have removed all subheadings in the Discussion.
3. Line 322 - Please temper assertions of primacy by adding ‘to the best of our knowledge’ or similar.
   - Response: “To the best of our knowledge” was added to the intended sentence (line 469).
4. Line 340 - please define IPDMA at first use
   - Response: We changed IPDMA to individual participant data meta-analysis in the introduction (line 168) and in the discussion (line 450).

**Figures:**
When a p value is given, please specify the statistical test used to determine it in the respective figure legend (including those in Supporting Information files).

- Response: The figures, included those in the Supporting Information files, have been edited accordingly.

**Tables:**
When a p value is given, please specify the statistical test used to determine it in the respective table legend (including those in Supporting Information files).

Please define all abbreviations used in the table legend of each table  (including those in Supporting Information files).

- Response: All abbreviations used have been defined under the tables and the statistical tests used have been included in the table legends, both in the manuscript file and the Supporting Information files.

**References:**
Please ensure that journal name abbreviations match those found in the National Center for Biotechnology Information (NCBI) databases, and are appropriately formatted and capitalised.
Please also see

- Response: The references have been formatted according to the suggested style and the journal abbreviations have been matched to those in the NCBI databases.

**Supplementary files:**

Please see our supporting information guidelines.

- Response: The Supplementary files have been edited according to the guidelines.

**COMMENTS FROM THE REVIEWERS:**

**Reviewer #1: Statistical review, James Wason**

This paper reports a RCT comparing low-dose aspirin to placebo for reducing pre-term birth. The methods and results are well reported. I have only some minor comments.

We thank the reviewer for the thorough review of the statistics and the comments to improve the manuscript. Please find our answers and a description of the changes below.

1. Abstract - I am unsure of whether featuring the subgroup analysis predominantly in the abstract is appropriate given that it is described as hypothesis generating and it is one of several subgroup analyses done.

- Response: We removed the results from the mentioned subgroup analysis from the Abstracts section (line 106-109).

2. Methods, outcomes - I would recommend that the paper lists all secondary outcomes in the main paper (although if complex the full definitions could be kept in the supplementary material).
   - Response: We have added the secondary outcomes reported in the main paper to the Methods section (line 226-229, line 233-243). The full definitions are provided in the supplementary material to avoid the text becoming too wordy.
3. Statistical analysis, Line 201 - I would recommend clarifying this is two-sided alpha.
   - Response: The suggested clarification has been made (line 267).
4. Statistical analysis, I would recommend that the method used to get relative risk reduction is mentioned here. If any covariates were adjusted for in the analysis, I would mention them here.
   - Response: The method used for calculation of the relative risks has now been described in the lines 273-280. No covariates were adjusted for in the analyses (line 282).
5. Statistical analysis, for the Bayesian analysis there is not enough information given - presumably an informative prior was used corresponding to 36% and 23% in the two arms, but how informative (i.e. what Beta distribution was used). Although in the SAP I would summarise it in the main paper.
   - Response: We have added additional information on the Bayesian analysis to the manuscript (line 283-285).
6. Statistical analysis, I would add what subgroups were pre-specified.
   - Response: The pre-specified subgroups have been defined in the methods section (line 287-292).
7. Line 256 -  perhaps add 'indicating no strong evidence of difference between arms' after the Bayes factor result?
   - Response: As suggested, we added “indicating no strong evidence of difference between arms” after the Bayes factor results (line 347).
8. Line 273 - I would edit this to 'no significant differences'.
   - Response: We altered the suggested sentence by adding “no significant differences” (line 369).
9. Methods/Results - I note the protocol mentions an economic analysis but this is not included here. It might be good to somewhere say that this will be reported elsewhere (or that it was planned for but not done).
   - Response: The planned economic analysis as mentioned in the protocol is currently in its final stage and will be reported elsewhere. This has been clarified in the methods section (line 298-299).

**Reviewer #2**: Nicely done paper. Unfortunately, as you point out, the study was underpowered to make a firm finding.

1. One question, if you did statistical re-analysis with the projected preterm delivery rate of 35% and risk reduction of 36% would your study show statistical/possible clinical significance?
   - Response: We thank the reviewer for this question. We found a preterm birth rate of 25.4% in the placebo group. In case of a hypothetical reduction in preterm birth of 36%, this would result in a preterm birth rate of 0.254x0.64=16.3% in the aspirin group (n= 31 out of 194 in the aspirin group). This corresponds with an RR of 0.63 (95% CI 0.42-0.94, p = 0.024). This would be statistically as well as clinically significant. However, these are not the observed results.

In the observed results, the preterm birth rate was 21.2% in the aspirin group and 25.4% in the placebo group (RR 0.83, 95% CI 0.58-1.20). If future larger trials find this reduction of approximately 17% to be statistically significant, we believe this will also be of clinical significance. Considering the magnitude of the disease burden of preterm birth, even small reductions in preterm birth will improve pregnancy outcomes worldwide.

**Reviewer #3:** This RCT of women with a prior SPTB comparing low dose aspirin (80mg) to placebo in women with  a history of preterm birth.  The project was well conducted with appropriate provision for masking and ascertaining outcomes. The paper is well written and the conclusions are well supported.  Similarly they adhere to the CROWN guidance and provide rich supplementary materials. The following comments are designed to improve the manuscript.

We thank the reviewer for their careful review of the manuscript and valuable comments. Please find a step-by-step response below.

Major:

1. The largest issue with this trial is the exceedingly ambitious power analysis. They assume a baseline rate from a study conducted with an American predominantly African-American population of whom almost have had 2 prior preterm deliveries. On top of this they assume an effect size of 35% when the prior literature had suggested a 10 to 20%.  Finally they set the power at 80%. It is therefore not surprising that the outcome is a trial in which the OR is directionally correct but not statistically significant. This is compounded by the fact as they describe there is a lack of parity in the two groups with the Aspirin group having higher risk.
   - Response: The reviewer is correct that the power analysis performed is ambitious. The largest issue with the underpowered sample size is that we cannot draw firm conclusions based on the findings. We have addressed this matter in the discussion.
2. Throughout the manuscript- p-Values are not kept to 2 significant digits.
   - Response: We have changed all p-values to 2 significant digits throughout the manuscript.
3. Preterm birth is the primary outcome yet the method of deriving this outcome is not clear established. Was a first trimester ultrasound required?  How were discrepancies in LMP and due date by ultrasound handled.
   - Response: In the Netherlands, the due date is determined by measurement of the fetal crown-rump length during a first trimester ultrasound between 8^+4^ and 12^+6^ weeks of gestation, ideally between 10^+0^ and 12^+6^ weeks. We have added a short description on the determination of the due date in the methods section (line 253-254).

Minor:

1. Introduction: You do not need to state that "The remaining 65%" as this is inferred from the prior sentence.
   - Response: We have altered the sentence and now only mention the proportion of spontaneous preterm births (line 156-158).
2. Intervention:  UMC and AMC are unclear abbreviations.
   - Response: We have written the full name of the centres in the text (line 215-216).
3. Methods: " The most important"  Should be removed.  This is a value judgement.
   - Response: We have altered the section on the secondary outcomes and have removed “the most important” as description of the composite of poor neonatal outcome (line 229-233).
4. Results:  "The rate of women .... (n=14 , 7.2%)  This number disagrees with the table that reports 13.  Please decide which is correct.
   - Response: Thank you for pointing out this irregularity. In the aspirin group, there were 13 women with one mid-trimester feral loss and 1 with multiple mid-trimester fetal losses. Therefore, there were 14 (7.2%) women with one or more mid-trimester fetal losses in the aspirin group. We have clarified this in the manuscript (line 326).
5. Table 1: please define education level and "quitted" should be quit or prior
   - Response: A definition of education levels was provided in the footnotes below table 1 (line 336-338). Furthermore, smoking “quitted” was changed to quit in Table 1.

**Reviewer #4**: I have read this paper in detail , now several times. It is an excellent  randomised controlled trial where the authors have faced the recurrent challenges of a less than expected outcome rate and therefore as they say the study in the final analysis is underpowered. Also the study demonstrates that randomisation does not result in perfectly balanced groups. The baseline imbalances were all in favour of the placebo group and therefore this could mask the true effectiveness of the aspirin. I consider the study outstanding partly because of the way the authors have discussed and interpreted it. In particular they articulate very clearly the  ambiguity of the results of their trial. This should serve as a model discussion piece.

I would just ask for one minor revision; given the  results, the underpowered nature of the study, the complexity of the arena and other challenges, a brief paragraph on the way forward with further investigations should be offered.

Response: We thank the reviewer for the appreciation of our manuscript. We have added a paragraph on our future perspective for research at the end of the discussion (line 483-493).
