# Supplementary material for: Evaluation of low-dose aspirin in the prevention of recurrent spontaneous preterm labour (the APRIL study): A multicentre, randomised, double-blinded, placebo-controlled trial
Source: PLoS Med. 2022 Feb 1;19(2):e1003892. doi: 10.1371/journal.pmed.1003892 (PMC8806064; doi:10.1371/journal.pmed.1003892)
Supplement: Attachment — Submitted filename: APRIL Response editors no. 2 20211125.docx [file pmed.1003892.s013.docx]

Amsterdam, November 2021

Response to the editors

We very much appreciate the opportunity to publish our manuscript in PLOS Medicine. We have made the requested changes to the manuscript. A point-by-point response to the comments is presented below.

- **In your Data Availability Statement, please provide some further detail as to the AMC policy on data sharing and how data will be made available to those who enquire.**
  As can be found in the metadata of the submission system, we have updated our Data Availability Statement as follows:
  Data are owned by Amsterdam University Medical Centers, location Academic Medical Center (AMC), that supports data sharing. For enquiries on data sharing, a methodologist from the trial centre within the Dutch Consortium for Healthcare Evaluation and Research in Obstetrics and Gynaecology (NVOG Consortium) may be contacted via [trialbureau@zorgevaluatienederland.nl](mailto:trialbureau@zorgevaluatienederland.nl). The de-identified data underlying this study will be made available for research purposes. The requestors are required to sign a data sharing agreement to ensure appropriate use of the data, and to ensure data confidentiality and security.
- **On line 136, the term "trend" is used to refer to a nonsignificant P value. The term trend should be used only when the test for trend has been conducted. Please revise accordingly.**
  We have removed the word trend and have altered the respective bullet point from “What Did the Researchers Do and Find?” as follows:
  The preterm birth rate was slightly lower in the aspirin group (21.2%) as compared to the placebo group (25.4%), especially among women with good adherence to medication (19.2% in the aspirin group versus 24.8% in the placebo group). These differences were not statistically significant.
- **Citations should be preceding punctuation, please update throughout.**
  We have changed this throughout the manuscript.
- **Please remove the ‘Competing interests’, ‘Financial Disclosure Statement’, and ‘Data availability’ sections from the end of the main text; this information will be captured as metadata based on your responses to the submission form questions (so please ensure all relevant information is included in your answers).**
  **Similarly, please remove the ‘Ethics approval’ section from the end of the main text as this information is already included in the methods section.**
  We have removed all the mentioned statements from the manuscript file as they are indeed mentioned in the submission form questions.
- **Table 3: Please confirm the meaning of the asterisk in table 3 or remove if it was included unintentionally.**
  The meaning of the asterisk has been added to table 3.
- **Table S2 and Appendix S4: Please replace the term “compliance” with “adherence” where it is used to refer to treatment adherence.**
  The term compliance was changed to adherence in Table S2 and Appendix S4.
